# Development of a Smartphone-Based Optical Device to Measure Hemoglobin Concentration Changes for Remote Monitoring of Wounds

**DOI:** 10.3390/bios11060165

**Published:** 2021-05-21

**Authors:** Kacie Kaile, Christian Fernandez, Anuradha Godavarty

**Affiliations:** Optical Imaging Laboratory, Florida International University, Miami, FL 33174, USA; kkail001@fiu.edu (K.K.); cfern340@fiu.edu (C.F.)

**Keywords:** near-infrared, smartphone, non-contact imaging, single value decomposition, signal-to-noise ratio, vascular occlusion test, hemoglobin concentrations, oxy-hemoglobin, deoxy-hemoglobin

## Abstract

Telemedicine (TM) can revolutionize the impact of diabetic wound care management, along with tools for remote patient monitoring (RPM). There are no low-cost mobile RPM devices for TM technology to provide comprehensive (visual and physiological) clinical assessments. Here, a novel low-cost smartphone-based optical imaging device has been developed to provide physiological measurements of tissues in terms of hemoglobin concentration maps. The device (SmartPhone Oxygenation Tool—SPOT) constitutes an add-on optical module, a smartphone, and a custom app to automate data acquisition while syncing a multi-wavelength near-infrared light-emitting diode (LED) light source (690, 810, 830 nm). The optimal imaging conditions of the SPOT device were determined from signal-to-noise maps. A standard vascular occlusion test was performed in three control subjects to observe changes in hemoglobin concentration maps between rest, occlusion, and release time points on the dorsal of the hand. Hemoglobin concentration maps were compared with and without applying an image de-noising algorithm, single value decomposition. Statistical analysis demonstrated that the hemoglobin concentrations changed significantly across the three-time stamps. Ongoing efforts are in imaging diabetic foot ulcers using the SPOT device to assess its potential as a smart health device for physiological monitoring of wounds remotely.

## 1. Introduction

### 1.1. Diabetes Health Care and Diabetic Foot Ulcers (DFUs)

Complications from diabetes mellitus (DM) are increasing [1], making DM a major global health problem [2,3,4]. According to the International Diabetes Federation (IDF), the USA has the third most affected population globally, with ~30.3 million cases in 2018 [5]. The lifetime risk for developing diabetic foot ulcers (DFUs) is 25% in patients with DM [6]. It is also reported that every 30 s, one lower limb amputation occurs in patients with DM worldwide [7]. North America has a prevalence of 13% of DFU cases [8], with over half of patients being at high risk and 1 in 10 having an amputation during their lifetime. Complications of DFUs occur when monitoring is infrequent and often results in serious sequelae like amputation or even death [9]. Without effective self-care, people with DFUs are at risk of prolonged healing times, hospitalization, amputation, and reduced quality of life. Despite these consequences, compliance with DFU self-care remains low. Telemedicine (TM) care is a promising solution for DFU management as the results are comparable with the typical standard of care (SOC) [10].

### 1.2. Telemedicine (TM) of DFUs 

The cornerstone of the TM systems for DFU care is the clinical assessment of digital photographic images from high-quality digital cameras or 3D cameras (portable, hand-held)—primarily used by health professionals and not patients. These images can reliably capture and measure ulcer size (or area) across healing. The use of TM was associated with significantly fewer amputations, reduced healing times for hard-to-heal ulcers [11] and is as safe and effective as SOC during post-operative wound care [11]. Although TM provided validity and reliability findings comparable to SOC, all TM systems investigated are expensive, require highly technical equipment, and are not readily accessible in remote settings [12]. Hence there is a need to apply low-cost mobile health (mHealth) technologies (using smartphones) into TM for daily clinical practice situations, including home-care nurses or self-care consultation by patients with financial or travel issues. 

### 1.3. mHealth and Smartphone Technologies in Diabetic Wound Care 

Nearly 60% of smartphone users gather health-related information on their phones, and 80% of physicians use smartphones and mHealth apps. Recently, researchers have developed smartphone-based apps for (i) 2D and 3D wound image analysis [13,14] to estimate and track wound size reduction, (ii) monitoring patient’s wound healing status from digital photographs, and (iii) select appropriate wound dressings [15] based on symptoms. However, the current mHealth DFU assessment using mobile phone images provides only minimal clinical assessment in terms of visual wound coloration, size (or area), and depth measurement characteristics [16]. They are inappropriate as stand-alone tools to remotely assess and treat follow-up DFU cases without additional clinical assessments (e.g., physiological assessments) [17]. 

Physiological assessment of DFUs is vital to augment the clinician’s visual assessment since sub-surface changes in wounds are not visible to the naked eye. The various physiological parameters measured to assess wound healing include tissue oxygenation, temperature, pH, and pressure. Smartphone adaptable thermal sensors (forward-looking infrared, FLIR) have been developed and used to obtain thermal maps of the wounds and assess their healing status [18]. An optical-fiber embedded smart bandage that measures the wound region’s pH and pressure has recently been developed [19]. Our research focuses on measuring the tissue oxygenation-based physiological parameter of the wounds using smartphone-based technology.

Oxygen supply to DFUs is a key limiting factor for successful healing due to increased demand for reparative processes such as cell proliferation, bacterial defense, angiogenesis, and collagen synthesis [20]. Hence, tissue oxygenation is one of the key physiological parameters used to assess the effectiveness of the treatment approach in improving oxygen supply to DFUs (and thus enhances healing rates). A smartphone-based near-infrared (NIR) optical imaging device was recently developed [21] to measure physiological changes in tissues (in terms of diffuse reflectance signals) across a wide area and without contact. A NIR add-on optical module including a 690 nm light-emitting diode (LED), LED drivers, and diffuser sheet (for uniform illumination) was attached to the smartphone. This add-on optical module was used in sync with the smartphone’s built-in camera to acquire diffuse reflectance images. The ability of the device to measure different reflectance changes (at 690 nm) was demonstrated via occlusion studies on control subjects [20]. Unlike diffuse reflectance measurements, tissue oxygenation measurements (in terms of oxy- and deoxy-hemoglobin concentrations) are clinically relevant when assessing wounds. Hence, the focus of the current study was to develop a smartphone-based NIR device capable of measuring tissue oxygenation changes in terms of oxy- and deoxy-hemoglobin concentration maps as a non-contact wide-area imaging approach. 

In this work, the development of our smartphone-based tissue oxygenation measuring device (smartphone oxygenation tool—SPOT) is described. The SPOT device consists of an add-on NIR optical module, a smartphone, and a custom-developed smartphone app that automates the data acquisition process. The optimal imaging conditions in terms of imaging heights and device settings are determined from signal-to-noise ratio studies. The feasibility of the SPOT device to map for oxy- and deoxy-hemoglobin changes in response to a standard vascular occlusion test is demonstrated with and without the application of noise-filtering techniques.

## 2. Materials and Methods

The SPOT instrumentation, related app, and data analysis to obtain tissue oxygenation maps are described in this section. The optimal signal-to-noise ratio (SNR) of the device and its feasibility to obtain tissue oxygenation changes in response to vascular occlusion test is determined. 

### 2.1. Smartphone Oxygenation Tool (SPOT) Instrumentation 

The first version of the SPOT device could obtain diffuse reflectance images at a single wavelength (690 nm) [21]. Herein, the SPOT device was modified to obtain diffuse reflectance images at multiple wavelengths (690, 810, 830 nm) and is further used to determine oxy- and deoxy-hemoglobin maps. The details of SPOT’s hardware, software, and data analysis procedures are described below.

#### 2.1.1. Hardware 

**Figure 1:**
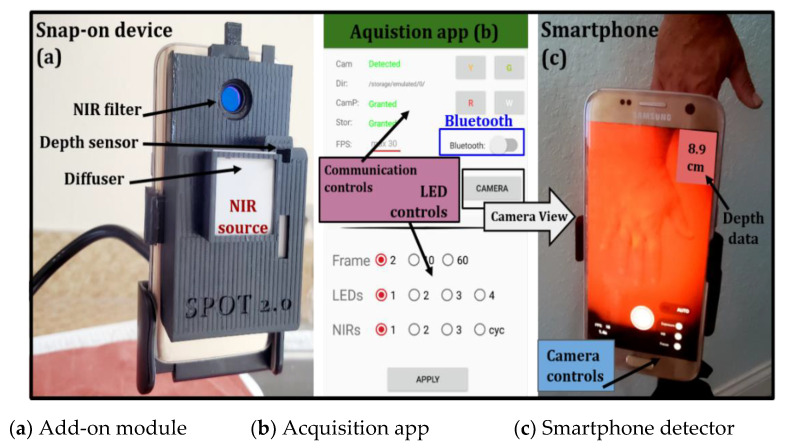
(**a**) Smartphone oxygenation tool (SPOT) hardware consisting of the add-on optical module clipped onto the smartphone; (**b**) Automated data acquisition app of the SPOT device that synchronizes the near infrared (NIR) light source and the smartphone’s detector; (**c**) Display screen of the smartphone with camera controls and image depth display, along with real-time diffuse reflectance signals.

The SPOT device constitutes an add-on optical module, smartphone, and related app that automated data acquisition (Figure 1). The add-on optical module includes the source and optical components required at the source and detector end. The near-infrared (NIR) source was developed using four multi-wavelength light-emitting diodes (LEDs). These surface-mounted LEDs (SMT690D/810/830) illuminate a wide area without contact. The custom-developed LED driver multiplexes these wavelengths at 3.2 Hz. A Bluetooth chip (HC-05) is used to communicate with the add-on optical module and control the LEDs. A diffuser sheet (LPNIRE, 600–1100 nm) is placed at the source end for uniform illumination intensity. The NIR light emitted by the LEDs onto the imaged surface is detected by the internal camera of the smartphone as a diffuse reflectance signal. A NIR long pass (LP) filter (LP645–13.25 mm) used in conjunction with a smartphone camera allows only the NIR signals to be detected. A distance sensor (VL53LOX) is also incorporated into the add-on optical module to detect imaging depths. The smartphone powers the add-on optical module through a universal serial bus (USB). The material cost of the add-on optical module (excluding the smartphone) is a few hundred dollars, making it a low-cost device. 

#### 2.1.2. Software

**Figure 2:**
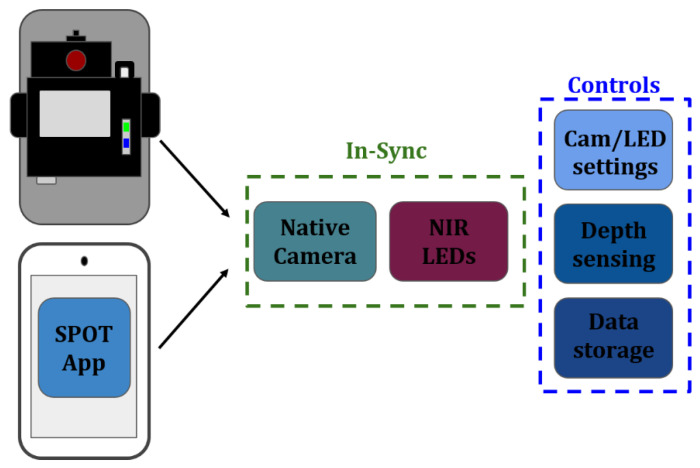
The add-on optical module combined with the custom SPOT app creates a unique platform syncing and controlling the internal hardware of the smartphone with the external hardware and storage permissions.

The SPOT hardware is controlled via a custom app that is developed in Android Studio (Java). The custom app communicates with the add-on optical module to control: (i) the number of LEDs to be turned on during imaging studies, (ii) the wavelength(s) of these LEDs that it will illuminate the tissue with (e.g., 690, 810, and/or 830 nm), and (iii) sync the source end with the smartphone’s internal camera to control the number of frames acquired, operating at a rate of 30 frames per second. The app also controls the smartphone’s internal camera settings in terms of ISO (International Organization for Standardization), focusing, white balance, and exposure (digital aperture). In addition to syncing the NIR source with the smartphone camera and controlling its internal settings, the app connects the distance sensor to provide a readout display of the imaging depth directly onto the camera view. The features that the SPOT app syncs and controls are shown in Figure 2. Development of the acquisition software syncs the external hardware (NIR LEDs/depth sensor) with internal smartphone hardware (camera/camera settings) and stores data on the smartphone for further processing. 

#### 2.1.3. Data Acquisition

**Figure 3:**
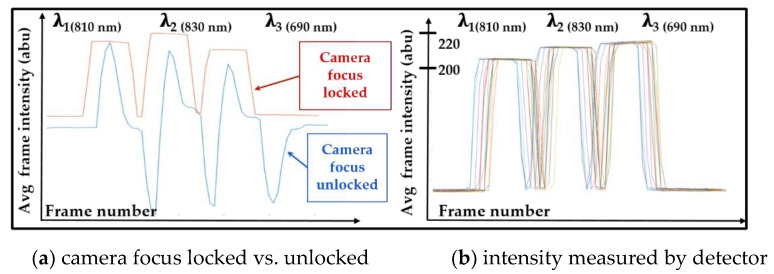
(**a**) The average diffuse reflected intensity across frames from the 1 s video file containing data across the three multiplexed wavelengths, with camera focus locked and unlocked. (**b**) The average diffuse reflected intensity across the frames from the 1-s video file with the camera focus locked and after optimizing the input optical powers across the three wavelengths. The multiple lines are across 20 repetitions.

The smartphone camera collected diffuse reflectance signals as short (~1 sec) video files (30 fps), and these video files were transferred to a computer for further processing. The diffuse reflectance images related to each illuminated wavelength (810, 830, and 690 nm) were extracted from average intensity plots that were distinctly different for each wavelength. During data acquisition, it was observed that the smartphone detector inherently executed focusing corrections when the NIR source multiplexed across the different wavelengths (as shown in Figure 3a, as blue color plot). Hence, the focus of the smartphone camera was locked (by the custom app) during imaging to obtain multiple data frames per wavelength with minimal deviation in the measured intensity (with no change in camera focus), as shown by the red color plot in Figure 3a. Additionally, the optical powers at each NIR wavelength were optimized to ~5 mW for 690 nm, ~28 mW for 810 nm, and ~30 mW for 830 nm (measured at point-blank by an optical power meter). These optical powers were chosen such that the detected intensity of the diffuse reflected signal was similar across the three wavelengths (shown in Figure 3b) with a maximum standard deviation of ±1.4 a.b.u at 690 nm, ±0.4 a.b.u at 810 nm, and ±0.9 a.b.u. at 830 nm across 20 multiplexed repetitions. The noticeable shift in intensity across the 20 multiplexed repetitions was due to hardware lag time. Hence, the smartphone camera was always turned on before the beginning of LED multiplexing. During this period, the ambient intensity images with LEDs turned off (I (x, y, λi, LED off)) were also measured. 

### 2.2. Data Analysis 

The SPOT device captured diffuse reflectance signals at multiple wavelengths (I_λi_ (x, y)) for tissue oxygenation analysis. This analysis was achieved using a Modified Beer Lamberts Law (MBLL) approach at two wavelengths (690 and 830 nm) [21,22]. The major chromophore of interest was hemoglobin, and knowing the absorption properties are significantly different when hemoglobin is bound with oxygen (oxy-hemoglobin) compared to unbound or reduced hemoglobin (deoxy-hemoglobin), effective concentrations of each (∆HbO and ∆HbR) can be estimated. The 690 and 830 nm wavelengths are typically used to detect the deoxy- and oxy-hemoglobin changes since they have high molar extinction coefficients (ɛ^λ^ chromophore) associated with oxy- and deoxy-hemoglobin, respectively [23,24,25,26]. Effective concentrations of oxygenation were achieved via changes in absorption with reference to a calibration image (I_ref_ _λi_ (x, y)). Calibration images were obtained at each wavelength (690 and 830 nm) using a uniformly diffusing white sheet. The dark noise (I_D_(x, y)) was obtained from the smartphone as the intensity measured with no light entering the camera. The optical density at each wavelength (∆OD (x, y, λi)) was estimated as the ratio of measured diffuse reflectance intensity compared to the calibration image given in Equation (1). Effective concentrations of oxy-hemoglobin (∆HbO) and deoxy-hemoglobin (∆HbR) were estimated from the measured optical densities given in Equations (2) and (3), respectively. The term L × B in Equations (2) and (3) correspond to the unknown values of the optical path length factor (L) and inter-optode geometry (B), which cannot be defined in non-contact NIR imaging. Hence oxy- and deoxy-hemoglobin concentrations are expressed as effective rather than absolute values.

(1) $$\Delta \mathrm{OD}\left(x,y,\lambda_{i}\right)=-\log\frac{I_{\lambda_{i}}\left(x,y\right)-I_{D}\left(x,y\right)}{I_{\mathrm{ref}\lambda_{i}}\left(x,y\right)-I_{D}\left(x,y\right)}$$

(2) $$\;\;\;\Delta \mathrm{HbO}\;\left(x,y\right)=\frac{\left\{\varepsilon_{\mathrm{HbR}}^{\lambda_{1}}\Delta {\mathrm{OD}}^{\left(x,y,\lambda_{2}\right)}-{\;\varepsilon }_{\mathrm{HbR}}^{\lambda_{2}}\;\Delta {\mathrm{OD}}^{\left(x,y,\lambda_{1}\right)}\right\}}{\left\{\left(\varepsilon_{\mathrm{HbR}}^{\lambda_{1}}\varepsilon_{\mathrm{HbO}}^{\lambda_{2}}-{\;\varepsilon }_{\mathrm{HbR}}^{\lambda_{2}}\varepsilon_{\mathrm{HbO}}^{\lambda_{1}}\right)\mathrm{LB}\right\}}$$

(3) $$\Delta \mathrm{HbR}\;\left(x,y\right)=\frac{\left\{\varepsilon_{\mathrm{HbO}}^{\lambda_{2}}\;\Delta {\mathrm{OD}}^{\left(x,y,\lambda_{1}\right)}-{\;\varepsilon }_{\mathrm{HbO}}^{\lambda_{1}}\;\Delta {\mathrm{OD}}^{\left(x,y,\lambda_{2}\right)}\right\}}{\left\{\left(\varepsilon_{\mathrm{HbR}}^{\lambda_{1}}\varepsilon_{\mathrm{HbO}}^{\lambda_{2}}-{\;\varepsilon }_{\mathrm{HbR}}^{\lambda_{2}}\varepsilon_{\mathrm{HbO}}^{\lambda_{1}}\right)\mathrm{LB}\right\}}$$

### 2.3. Maximizing the Signal-to-Noise Ratio (SNR)

**Figure 4:**
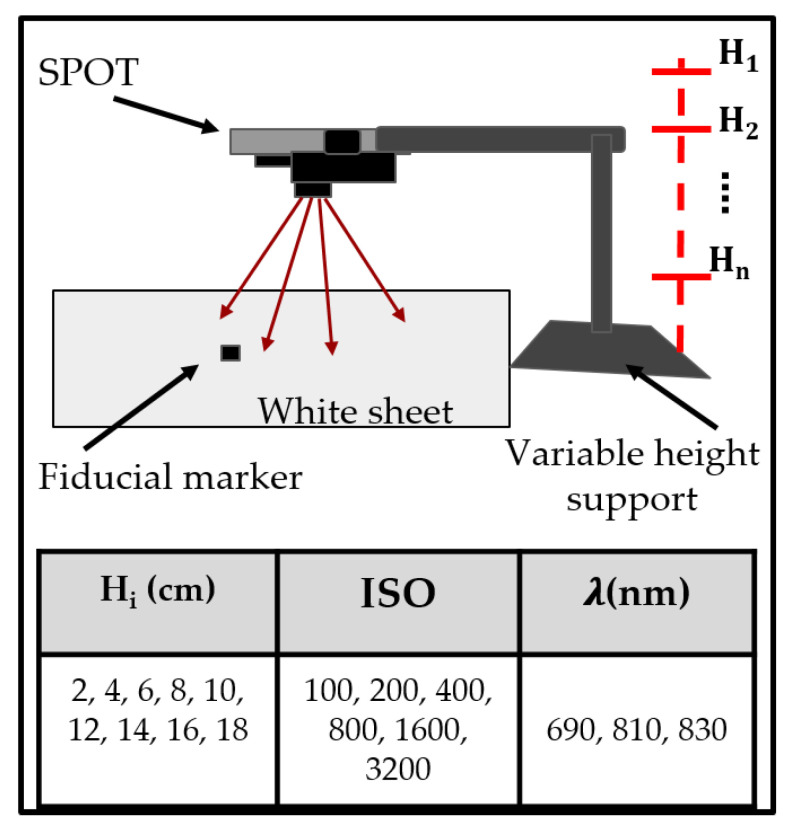
Experimental set-up for signal-to-noise ratio (SNR) studies using SPOT. Various heights are tested at all available smartphone camera’s ISOs (International Organization for Standardization) across the three NIR wavelengths.

The SPOT device’s signal-to-noise ratio (SNR) to acquire NIR images at three wavelengths (690, 810, and 830 nm) was determined under various imaging conditions. The SNR was maximized by optimizing the imaging distance (i.e., the distance between the source and imaging plane) and camera controls (i.e., ISO or International Organization for Standardization, which relates to the sensitivity of the camera) of the SPOT device. For medical imaging applications, SNRs above 20 dB are good, and when these exceed 50 dB, this is considered excellent [27,28]. Herein, diffuse reflectance images of the calibration white sheet were obtained under varying imaging depths between 2–18 cm and camera ISOs ranging from 100–3200. A black fiducial marker was used as a reference to estimate the theoretical imageable area achievable at the optimal SNR imaging height. The experimental set-up is shown in Figure 4. 

The SNR was calculated from diffuse reflectance signals obtained with the LED source turned on and turned off at various locations (Loc #) or regions of interest (ROIs), as given in Equation (4).

(4) $${\mathrm{SNR}}_{\lambda_{i}}(x,\;y,\;\mathrm{Loc}\;\#)=\;20\log[\frac{\left(I_{\lambda_{i}}\left(x,y,\;\mathrm{LED}\;\mathrm{on}\right)-I_{\lambda_{i}}\left(x,y,\;\mathrm{LED}\;\mathrm{off}\right)-I_{D}\left(x,y\right)\right)}{\mathrm{std}\left(I_{\lambda_{i}}\left(x,y,\;\mathrm{LED}\;\mathrm{on}\right)\right)}]$$

**Figure 5:**
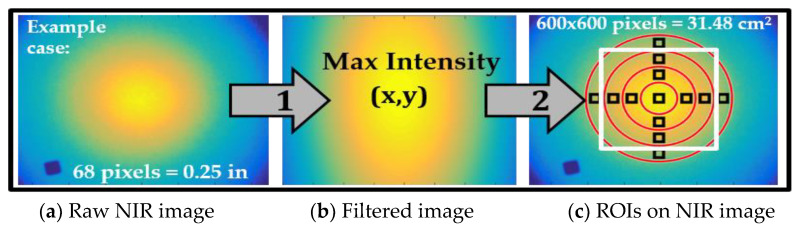
SNR calculations for 13 regions of interest (ROIs): the center ROI determines optimal imaging heights based on the maximum intensity in each NIR image at a given camera’s ISO, imaging height, and source wavelength (parameters shown in Figure 4). The remaining ROI SNR values are used to estimate the theoretical area of analysis with reference to the fiducial marker.

For each imaging condition, the center of the NIR source was identified within diffuse NIR images by applying a Gaussian filter and auto locating the point of maximum intensity. An ROI was generated directly at this maximum intensity or source beam center as a 25 × 25 boxed pixel region. Twelve additional ROIs (25 × 25) were automatically selected 90° apart to span the concentric circles separated by 100 pixels and centered with respect to the maximum intensity point, as shown in Figure 5. The SNR calculated at the center ROI was used to determine an acceptable imaging height range at each ISO setting of the smartphone’s camera. The SNRs calculated at the remaining ROIs were, in turn, used to estimate the maximum imaging area above a cut-off SNR value used to acquire tissue oxygenation maps. The optical imaging height and ISO with maximum SNR were determined at each wavelength, and the corresponding area with SNR ≥20 dB was estimated. These optimized parameters were used during in vivo occlusion studies. 

### 2.4. In Vivo Feasibility Study: Vascular Occlusion Test

The SPOT device was developed to map for tissue oxygenation changes in terms of ∆HbO and ∆HbR. Herein, a standard vascular occlusion test (VOT) [29] was performed to demonstrate the feasibility of our smartphone-based device to measure these hemoglobin concentration changes in control subjects. In this approach, a blood pressure cuff was used to induce occlusion and determine the hemoglobin changes before, during, and after the occlusion. 

**Figure 6:**
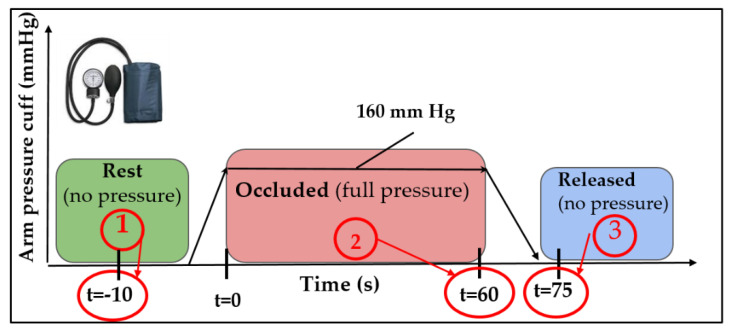
Vascular occlusion test of the dorsal of the hand using SPOT device, where diffuse reflectance images were acquired at the three-time stamps - (**1**) rest, (**2**) occlusion, and (**3**) released.

Three control (>18 years) subjects without any known pre-existing conditions were recruited for this Internal Review Board (IRB) approved study. SPOT device was mounted ~10 cm above the dorsal of the hand, and diffuse reflected NIR images were acquired (at ISO 800 camera settings) at 690 and 830 nm wavelengths. Subjects were seated and asked to breathe normally throughout the study. Calibration images were obtained by placing the diffusing white sheet on the tissue surface. The first point of imaging was conducted at rest, while the blood was flowing normally through the arm and the cuff applied no pressure. The arm pressure cuff was then increased to 160 mmHg and held constant for 60 s, at which point the second set of images were acquired, while blood flow was restricted. Immediately following this, the pressure was released entirely from the arm cuff. The third point of imaging was performed 10 s following the full release of pressure when the blood flow was restored. The three imaging points are shown in Figure 6 (highlighted with red circles). The three-time points were chosen at complete rest, total occlusion, and immediate release to capture maximum contrast in tissue oxygenation. 

#### 2.4.1. Data Analysis

The dual-wavelength diffuse reflectance NIR images in response to VOT were processed using the MBLL model in Matlab. As a first step, the imaging area with SNR ≥ 20 dB was demarcated before obtaining the ∆HbO and ∆HbR maps at each timestamp. Noise-filtering techniques were implemented, and their impact on hemoglobin maps was evaluated. The statistical significance of the differences in hemoglobin concentration maps across the three-time stamps was determined.

(a) Region of Interest (ROI) selection from SNR maps

Diffuse reflectance NIR images were converted into SNR maps (as described by Equation (5)), and the ROI with SNR ≥20 dB was determined.

(5) $${\mathrm{SNR}}_{\lambda_{i}}\;(x,\;y)=20\log\left[\frac{\left(I_{\lambda_{i}}\left(x,y,\;\mathrm{LED}\;\mathrm{on}\right)-I_{\lambda_{i}}\left(x,y,\;\mathrm{LED}\;\mathrm{off}\right)-I_{D}\left(x,y\right)\right)}{\mathrm{std}\left(I_{\lambda_{i}}\left(x,y,\;\mathrm{LED}\;\mathrm{on}\right)-I_{\lambda_{i}}\left(x,y,\;\mathrm{LED}\;\mathrm{off}\right)-I_{D}\left(x,y\right)\right)}\right]$$

(b) Noise Removal

**Figure 7:**
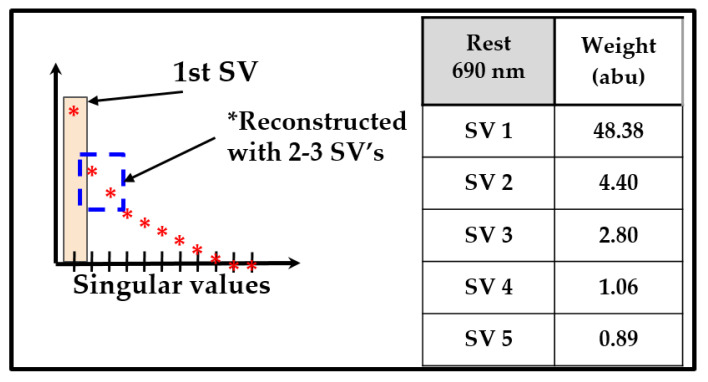
Singular values shown for a sample deconstructed NIR optical density at 690 nm and under rest conditions.

**Figure 8:**
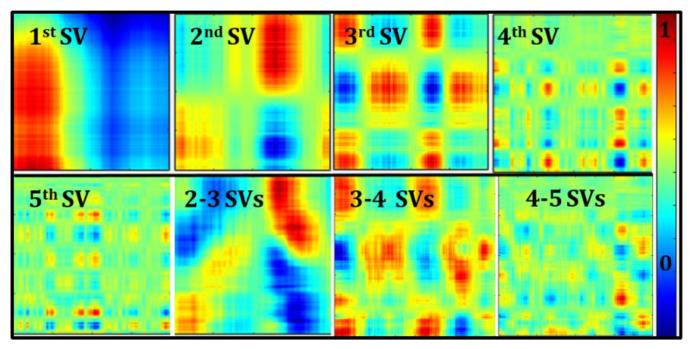
Two-dimensional (2D) spatial maps of effective oxy-hemoglobin (∆HbO) estimated by reconstructing the optical densities with individual singular values (SVs) between 1 and 5 and a combination of SVs. The above are sample data from one of the recruited subjects under rest conditions.

Single value decomposition (SVD) [30] was applied to the dual-wavelength optical density data (across the ROI selected based on the SNR maps). The first 3 singular values (SVs) accounted for more than 90% of the weight of SVs in the deconstructed image shown in Figure 7. The hemoglobin concentration (or tissue oxygenation) maps were then estimated using the various reconstructed optical densities and compared. Effective oxy-hemoglobin maps reconstructed using individual channels between 1–5 SVs are shown in Figure 8 as a sample case from one subject under rest conditions. Reconstruction of images using SVs 2–3, 3–4, and 4–5 are also shown in Figure 8. Removal of the first SV qualitatively removed the surface effects within oxygenation maps, likely due to the large specular reflectance in diffuse NIR images. Surface effects mask the underlying contrast in hemoglobin concentration between blood vessels and the surrounding tissues. The final ∆HbO and ∆HbR maps were constructed using SVs 2–3 since these singular values effectively differentiate the blood vessels compared to other SVs.

#### 2.4.2. Statistical Analysis 

**Figure 9:**
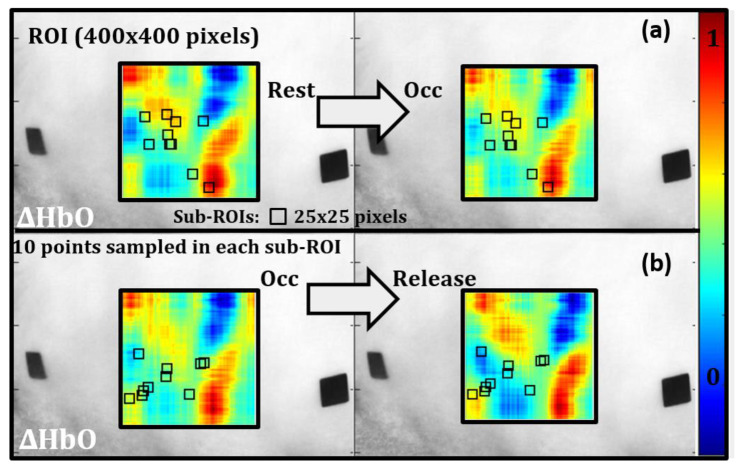
Ten sub-ROIs (25 × 25 pixels) were randomly selected to assess changes in oxygenation between (a) rest vs. occlusion and (b) occlusion vs. release. Within each sub-ROI, 10 points were chosen randomly to perform a statistical *t*-test between two consecutive timestamps.

A paired *t*-test was conducted to determine if there was a significant change in effective ∆HbO and ∆HbR in response to the applied stimulus using the blood pressure cuff. Ten locations or sub-ROIs (25 × 25 pixel regions) were randomly selected within the oxygenation map (similar across ∆HbO and ∆HbR maps) to assess the differences between rest vs. occlusion (statistically). A separate randomized set of 10 locations were selected to compare oxygenation differences between occlusion and release time stamps. An example case is shown for ∆HbO in Figure 9. Within each 25 × 25 sub-ROI, 10 points were sampled randomly, and a paired t-test (α = 0.05) was conducted for each sub-ROI (10) with the hypothesis that there was no significant change in oxygenation between the time points tested.

## 3. Results

### 3.1. Maximum SNR and Optimal Imaging Depths

The range of SNR values calculated across all ISO settings of the camera and the NIR wavelengths (690 and 830 nm) are given in Table 1. The maximum SNR measured for ISOs 100-3200 occurred at ISO 800 between 8–14 cm at both the NIR wavelengths (86 dB at 690 nm and 76 dB, respectively). The SNR values between the two wavelengths were similar across ISOs 100–800 but showed significant variations at ISO 1600 and 3200. For this particular Samsung device, ISO 1600 and 3200 exhibited the lowest SNRs in the measured signal and were not sufficient for NIR diffuse imaging. At the 8–14 cm range of imaging height and ISO 800 settings, the estimated imaging area with SNR ≥20 dB is given in Table 2. The imaging area was calculated using the 0.25 in^2^ black fiducial marker as the reference. Across the various imaging depths, the estimated imaging area (or field of view with SNR ≥20 dB) was higher at 8–10 cm heights. Hence, these image settings (ISO 800, imaging height between 8–10 cm) were used during in vivo feasibility studies. The hemoglobin concentration maps were constructed using diffuse reflectance data obtained at 690 and 830 nm in these preliminary feasibility studies. Hence, the SNR optimization results are only provided for these two wavelengths. 

### 3.2. In Vivo Vascular Occlusion Test (VOT) Feasibility Studies

The forearm comprises two main blood supplies stemming from the subclavical artery known as the axillary and brachial arteries. These extend down the lower arm as the radial and ulnar arteries and to the hand as the deep and superficial palmar arch. Common digital arteries extend from the arch to supply the fingers and thumb with a constant blood supply. In this total occlusion study, changes in oxygenation are expected to occur within the hand, corresponding to regions where this blood net extends through the dorsal of the hand. 

**Figure 10:**
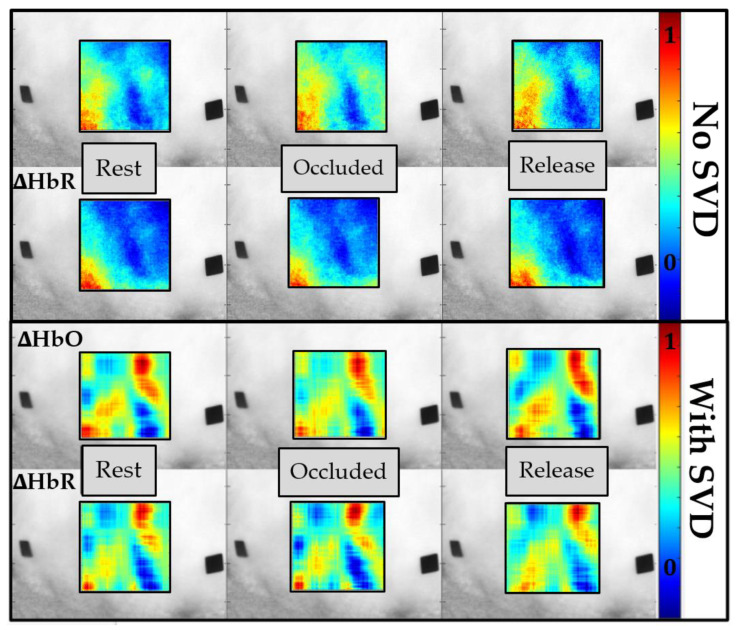
Effective oxy-hemoglobin **(**∆HbO) and deoxy-hemoglobin (∆HbR) maps in response to vascular occlusion are shown with and without the SVD-based noise removal technique. Black fiducial markers were placed on the dorsal of the hand of each subject for spatial reference. Pseudo-color oxygenation maps within the acceptable ROI (SNR>20 dB) are overlaid onto the grayscale NIR image with regions of red corresponding to higher concentrations and regions of blue corresponding to lower concentrations of the given parameter (∆HbO and ∆HbR). The plots with SVD noise filtration implemented employed 2–3 SVs during image reconstructions.

**Figure 11:**
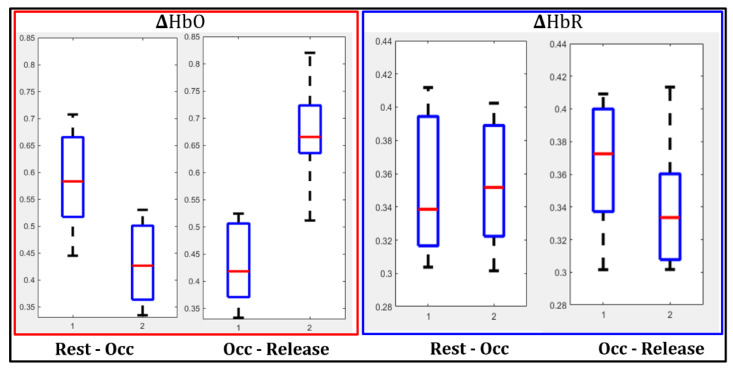
Boxplots for ∆HbO and ∆HbR are shown for rest vs. occlusion and occlusion vs. release in a single subject case and across the 10 randomly selected ROIs. The 10 random points within each ROI were averaged during this analysis. SVD noise filtering was applied during analysis.

Past VOT studies by other researchers demonstrated that oxy-hemoglobin decreased, and deoxy-hemoglobin increased during occlusion as oxygen was consumed for normal cell functions and not replaced by newly oxygenated blood flowing in [31]. In the current study using SPOT, the resulting tissue oxygenation maps across the standard occlusion are shown in Figure 10, with and without applying the noise removal technique (SVD). Qualitatively, a decrease in ∆HbO and an increase in the ∆HbR were observed during complete occlusion as seen at the perceived vessels. This change was apparent when quantified for a single subject’s case, represented as boxplots given in Figure 11. The drop in ∆HbO with occlusion and its increase with the release was distinct.

On the other hand, the decrease in ∆HbR post occlusion (or release) was distinct from the increased flow of oxygenated blood. In summary, as the pressure was released, ∆HbO increased, and ∆HbR decreased, as observed by past researchers [31]. This change in response to occlusion was noticeable when surface noise was removed using SVD. The significance in the extent of these changes was statistically evaluated using the paired *t*-test (as described in Section 2.4.2). 

Paired *t*-tests were conducted at 10 different sub-ROIs within the oxygenation maps (∆HbO and ∆HbR) between rest, occlusion, and release. The paired *t*-test results are given in Table 3 for each of the 10 locations sampled and for all 3 subjects, in terms of ∆HbO and ∆HbR between (i) rest vs. occlusion and (ii) occlusion vs. release.

Across the 3 subjects, there was a significant difference in ∆HbO and ∆HbR between the three timestamps in a majority of the 10 sub-ROIs sampled. However, the difference is not significant for all sub-ROIs, and this may be due to spatial changes in the vasculature. Since the ROIs were randomly selected and not specifically on the blood vessels where occlusion causes a change in oxygenation, it possibly impacts the total number of ROIs that showed a significant difference between rest-occlusion and occlusion-rest. Sub-ROIs chosen over the vasculature may show a very significant change, while sub-ROIs away from the vasculature may show little to no change. In the future, sampling may be more consistent by segmenting regions expected to correlate with the vasculature. However, the change measured at each sub-ROI was significant in the majority of the 10 locations sampled between time points across all subjects. In addition, the repeatability of these oxygenation changes in response to VOT within subjects will also be assessed as part of our future extensive validation studies. 

## 4. Discussion and Conclusions

A smartphone-based near-infrared spectroscopy (NIRS) system was developed as a SPOT device that can measure changes in oxy- and deoxy-hemoglobin concentrations across a wide field via non-contact imaging. The SPOT device is currently adaptable to an android based smartphone (Samsung Galaxy 7.0). The optimal camera settings included ISO 800 for maximum SNR, and the optical imaging depth ranged from 8–10 cm. The add-on optical module is adaptable to other smartphones, with minor changes in the device’s casing design. It aligns with the smartphone’s size and camera location for a snap-on fit.

Additionally, the optimal camera settings and image depths may be re-evaluated based on the internal camera of the smartphone. The SPOT app currently automates the data acquisition aspect of imaging such that the add-on optical module and the smartphone’s camera communicate effectively. The SPOT app is presently being expanded to allow for automated data extraction (by wavelength) and analysis to provide hemoglobin concentration maps.

The SPOT device’s ability to capture in-vivo hemoglobin concentration changes in response to vascular occlusion has been demonstrated as a feasibility assessment of the technology for tissue oxygenation mapping. While the VOT studies are a first step to observe if the smartphone can detect hemoglobin concentration changes during NIR imaging, there is a need for a systematic phantom study. Phantom studies will be carried out to assess the accuracy and sensitivity of the SPOT device to measure oxy- and deoxy-hemoglobin concentrations. In addition, repeatability studies (both phantom and VOT) will also be carried out to assess the precision of our SPOT device. In an ongoing pilot study, SPOT is also used to map for tissue oxygenation changes in diabetic foot ulcer (DFU) subjects across 4 weeks of treatment. Preliminary studies using SPOT were promising since they observed similar changes in the wound: background tissue oxygenation concentrations (as obtained using a commercial NIRS device) with the healing of a DFU. The accuracy of our SPOT device in tissue oxygenation measurements will also be assessed in comparison with the commercial NIRS device.

Many NIR-based optical techniques have been developed to assess oxygenation-based physiological changes in DFUs. Although these devices are either hand-held, low-cost, and/or non-contact commercial devices, they are not readily accessible for mHealth-based TM applications of DFUs. DFU wound care management requires frequent clinical visits that affect patients’ time, effort, finances, and/or travel limitations in remote settings. Non-compliance to regular care and follow-up of chronic DFUs can lead to hospitalization or amputations due to severe infections. TM/mHealth empowers people with DFUs in remote care away from the clinic. While smartphones are constantly used to provide a digital visual assessment of the wound status, they cannot serve as stand-alone devices to provide underlying physiological changes that manifest early on over visual changes, as obtained herein using SPOT. Even if the SPOT device may lack the sensitivity or accuracy of existing (bulky) commercial NIR-based devices for DFUs, its potential to impact a majority of DFU patients via a remote-care approach in the long term is more promising than sophisticated devices available only in interdisciplinary clinics or imaging centers. 

## 5. Patents

“Cellphone based tissue oxygenation measuring device” Inventors: A. Godavarty, K. Kaile, US Patent Publication No. 20200352515, Dec 2020 

## Figures and Tables

**Table 1:** Signal-to-noise ratio (SNR) results shown for 690 and 830 nm as the range of min-max values calculated for all ISOs. The min-max range of SNRs is across all heights (2–18 cm).

ISO	SNR Range: 690 nm	SNR Range: 830 nm
100	(43–80) dB	(36–73) dB
200	(40–81) dB	(46–73) dB
400	(18–85) dB	(34–75) dB
800 *	(25–86) dB *	(19–76) dB *
1600	(−2–75) dB	(−30–49) dB
3200	(−5–66) dB	(2–35) dB

* corresponds to the ISO with maximum SNR.

**Table 2:** The imageable area at varying imaging heights for each illuminated wavelength (690 and 830 nm). The camera’s ISO was 800, and the area was calculated of the region with SNR ≥20 dB.

Height (cm)	690 nm: Area (cm^2^)	830 nm Area: (cm^2^)
8 *	48.58 *	41.74 *
10 *	48.58 *	38.90 *
12	29.55	29.55
14	24.06	9.29

* Corresponds to imaging heights with maximum imageable area.

**Table 3:** Paired *t*-test showing the number of *p*-values < 0.05 across 10 sub-ROIs calculated for each subject between rest vs. occlusion and occlusion vs. release for ∆HbO and ∆HbR. The data represents the number of cases (total 10 cases) with *p* < 0.05 to demonstrate a significant difference.

Subject	∆HbO (rest-occ)	∆HbO (occ-release)	∆HbR (rest-occ)	∆HbR (occ-release)
1	10/10	9/10	6/10	8/10
2	8/10	10/10	7/10	10/10
3	7/10	8/10	6/10	9/10

## Data Availability

The data presented in this study are available on request from the corresponding author.

## References

[1] Lavery L.A., Peters E.J.G., Armstrong D.G. (2008). What are the most effective interventions in preventing diabetic foot ulcers?. Int. Wound J..

[2] Javanbakht M., Baradaran H.R., Mashayekhi A., Haghdoost A.A., Khamseh M.E., Kharazmi E., Sadeghi A. (2011). Cost-of-Illness Analysis of Type 2 Diabetes Mellitus in Iran. PLoS ONE.

[3] Yazdanpanah L., Shahbazian H., Aleali A.M., Jahanshahi A., Ghanbari S., Latifi S. (2016). Prevalence, awareness and risk factors of diabetes in Ahvaz (South West of Iran). Diabetes Metab. Syndr. Clin. Res. Rev..

[4] Ahmed M.A., Muntingh G.L., Rheeder P. (2017). Perspectives on Peripheral Neuropathy as a Consequence of Metformin-Induced Vitamin B12 Deficiency in T2DM. Int. J. Endocrinol..

[5] International Diabetes Federation (2017). Annual Report. https://www.idf.org/our-activities/advocacy-awareness/resources-and-tools/149:idf-annual-report-2017.html.

[6] Singh N., Armstrong D.G., Lipsky B.A. (2005). Preventing Foot Ulcers in Patients With Diabetes. JAMA.

[7] International Diabetes Federation (2005). Time to Act: Diabetes and Foot Care.

[8] Zhang P., Lu J., Jing Y., Tang S., Zhu D., Bi Y. (2017). Global epidemiology of diabetic foot ulceration: A systematic review and meta-analysis. Ann. Med..

[9] Wallace D., Perry J., Yu J., Mehta J., Hunter P., Cross K.M. (2019). Assessing the Need for Mobile Health (mHealth) in Monitoring the Diabetic Lower Extremity. JMIR MHealth UHealth.

[10] Smith-Strøm H., Igland J., Østbye T., Tell G.S., Hausken M.F., Graue M., Skeie S., Cooper J.G., Iversen M.M. (2017). The Effect of Telemedicine Follow-up Care on Diabetes-Related Foot Ulcers: A Cluster-Randomized Controlled Noninferiority Trial. Diabetes Care.

[11] Bolton L. (2019). Telemedicine Improves Chronic Ulcer Outcomes. Wounds Compend. Clin. Res. Pract..

[12] Bowling F.L., King L., Paterson J.A., Hu J., Lipsky B.A., Matthews D.R., Boulton A.J.M. (2011). Remote assessment of diabetic foot ulcers using a novel wound imaging system. Wound Repair Regen..

[13] Wang L., Pedersen P.C., Strong D.M., Tulu B., Agu E., Ignotz R. (2014). Smartphone-Based Wound Assessment System for Patients With Diabetes. IEEE Trans. Biomed. Eng..

[14] Sirazitdinova E., Deserno T. (2017). Abstract: Wound Imaging in 3D Using Low-Cost Mobile Devices. Informatik aktuell.

[15] Jordan S., McSwiggan J., Parker J., Halas G.A., Friesen M. (2018). An mHealth App for Decision-Making Support in Wound Dressing Selection (WounDS): Protocol for a User-Centered Feasibility Study. JMIR Res. Protoc..

[16] Bijan N., Jeong-Yeol Y. (2020). Digital health for monitoring and managing hard-to-heal wounds. Smartphone Based Medical Diagnostics.

[17] Van Netten J.J., Clark D., Lazzarini P.A., Janda M., Reed L.F. (2017). The validity and reliability of remote diabetic foot ulcer assessment using mobile phone images. Sci. Rep..

[18] van Doremalen R., van Netten J.J., van Baal J., Vollenbroek-Hutten M., van der Heijden F. (2019). Validation of low-cost smartphone-based thermal camera for diabetic foot assessment. Diabetes Res. Clin. Pr..

[19] Leal-Junior A., Guo J., Min R., Fernandezs A.J., Frizera A., Marques C. (2021). Photonic smart bandage for wound healing as-sessment. Photonics Res..

[20] Schreml S., Szeimies R., Prantl L., Karrer S., Landthaler M., Babilas P. (2010). Oxygen in acute and chronic wound healing. Br. J. Dermatol..

[21] Kaile K., Godavarty A. (2019). Development and Validation of a Smartphone-Based Near-Infrared Optical Imaging Device to Measure Physiological Changes In-Vivo. Micromachines.

[22] Kwasinski R., Fernandez C., Leiva K., Schutzman R., Robledo E., Kallis P., Borda L.J., Kirsner R., Perez-Clavijo F., Godavarty A. (2019). Tissue Oxygenation Changes to Assess Healing in Venous Leg Ulcers Using Near-Infrared Optical Imaging. Adv. Wound Care.

[23] Barker J.W., Panigrahy A., Huppert T.J. (2014). Accuracy of oxygen saturation and total hemoglobin estimates in the neonatal brain using the semi-infinite slab model for FD-NIRS data analysis. Biomed. Opt. Express.

[24] Barstow T.J. (2019). Understanding near infrared spectroscopy and its application to skeletal muscle research. J. Appl. Physiol..

[25] Xu R., Qiang B., Mao J. Near Infrared Imaging of Tissue Heterogeneity: Probe Design and Sensitivity Analysis. Proceedings of the 2005 IEEE Engineering in Medicine and Biology 27th Annual Conference.

[26] Pollonini L. (2018). Optical Properties and Molar Hemoglobin Concentration of Skeletal Muscles Measured In Vivo With Wearable Near Infrared Spectroscopy. IEEE Sensors J..

[27] Scientific Volume Imaging: Signal to Noise Ratio. https://svi.nl/SignalToNoiseRatio.

[28] Noise in Photographic Images. https://www.imatest.com/docs/noise/.

[29] Ganesan G., Cotter J.A., Reuland W., Cerussi A.E., Tromberg B.J., Galassetti P. (2015). Effect of Blood Flow Restriction on Tissue Oxygenation during Knee Extension. Med. Sci. Sports Exerc..

[30] Sadek R.A. (2012). SVD Based Image Processing Applications: State of The Art, Contributions and Research Challenges. Int. J. Adv. Comput. Sci. Appl..

[31] Boas D.A., Franceschini M.A. (2011). Haemoglobin oxygen saturation as a biomarker: The problem and a solution. Philos. Trans. R. Soc. A Math. Phys. Eng. Sci..

